# Deep learning methodology for predicting time history of head angular kinematics from simulated crash videos

**DOI:** 10.1038/s41598-022-10480-w

**Published:** 2022-04-20

**Authors:** Vikas Hasija, Erik G. Takhounts

**Affiliations:** 1 Bowhead (Systems & Technology), Washington, DC USA; 2 National Highway Traffic Safety Administration (NHTSA), Washington, DC USA

**Keywords:** Engineering, Biomedical engineering

## Abstract

Head kinematics information is important as it is used to measure brain injury risk. Currently, head kinematics are measured using wearable devices or instrumentation mounted on the head. This paper evaluates the deep learning approach in predicting time history of head angular kinematics directly from videos without any instrumentation. To prove the concept, a deep learning model was developed for predicting time history of head angular velocities using finite element (FE) based crash simulation videos. This FE dataset was split into training, validation, and test datasets. A combined convolutional neural network and recurrent neural network based deep learning model was developed using the training and validations sets. The test (unseen) dataset was used to evaluate the predictive capability of the deep learning model. On the test dataset, correlation coefficient obtained between the actual and predicted peak angular velocities was 0.73, 0.85, and 0.92 for X, Y, and Z components respectively.

## Introduction

In the United States, traumatic brain injury (TBI) is a serious public health issue. In 2014, about 2.87 million TBI related emergency department (ED) visits, hospitalizations and deaths occurred in United States^1^. Falls and motor vehicle crashes (MVC) were the first and second leading causes of TBI-related hospitalizations^1^. The lifetime economic cost of TBI, including direct and indirect medical costs, was estimated to be approximately $76.5 billion (in 2010 dollars)^2^. Given the cost and number of TBI cases, understanding the mechanism of brain injury and preventing them is critical. Researchers over the years have found head motion kinematics to be an important correlate to brain injuries. Many head/brain injury metrics that have been developed such as the head injury criterion (HIC)^3^, brain injury criterion (BrIC)^4^, rotational injury criterion (RIC)^5^ etc. all make use of head motion kinematics. HIC is based on linear accelerations and is part of vehicle safety regulation^6^, BrIC is based on angular velocities and RIC is based on angular accelerations. Measuring head kinematics is thus extremely important to understand the risk of brain injury.

Deep learning is a part of machine learning based on artificial neural networks and has been shown to be very effective in solving complex problems in the area of computer vision, natural language processing, drug discovery, medical image analysis, etc. Recently, deep learning models were used in brain injury biomechanics field as well. Wu et al.^7^ used American college football, boxing and mixed martial arts (MMA) datasets along with lab-reconstructed National Football League impacts dataset to develop a deep learning model to predict 95th percentile max principal strain of the entire brain and the corpus callosum along with fiber strain of the corpus callosum. Zhan et al.^8^ used kinematic data generated by FE simulations and those collected from on-field football and MMA using instrumented mouthguards and developed a deep learning head model to predict the peak maximum principal strain (MPS) of every element in the brain. Ghazi et al.^9^ developed a convolutional neural network (CNN) to instantly estimate element-wise distribution of peak maximum principal strain of the entire brain using two-dimensional images of head rotational velocity and acceleration temporal profiles as input to CNN model. Also, Bourdet et al.^10^ developed a deep learning model with linear accelerations and linear velocities from helmet tests as input to the model to predict maximum Von Mises stress within the brain.

In addition to predicting strains and stresses in the brain, deep learning models have also been developed to detect impacts to the head in American Football. Gabler et al.^11^ evaluated a broad range of machine learning (ML) models and developed a Adaboost based ML model to discriminate between head impacts and spurious events using 6DOF head kinematic data collected from a custom-fit mouthguard sensor. More recently, Raymond et al.^12^ used head kinematic data from instrumented mouthguards augmented with synthetic head kinematic data obtained from FE head impacts to detect impacts to the head using physics-informed ML model.

One common thread in these studies is that they use head kinematics data obtained from FE simulations/ wearable devices/ head instrumentation as input for their deep learning models to predict either strains in the brain or detect impact to the head.

Related to head kinematics, video analysis has also been used in the past. For example, Sanchez et al.^13^ evaluated laboratory reconstruction videos of head impacts collected from professional football games. The videos were generated from a high-speed camera recording at 500 frames per second. These videos were not used to predict or compute head kinematics but were analyzed to identify a time region of applicability (RoA) for head kinematics and for application to FE brain models to determine MPS and cumulative strain damage measure (CSDM)^14^.

The goal of this study was to evaluate the feasibility of deep learning methodology to predict time history of head angular kinematics directly from simulated crash videos and its applicability to controlled testing environments like National Highway Traffic Safety Administration (NHTSA) commissioned vehicle crash tests. As a proof of concept, a deep learning model was developed to predict time history of X, Y and Z-components of head angular velocity vector from FE based crash videos. Angular velocity has been shown to better correlate with brain strains as compared to angular accelerations^15^ and is used in brain injury criterion (BrIC) developed by NHTSA for assessing risk of TBI. For these reasons, predicting angular velocity time histories was chosen for this study, while corresponding angular accelerations could be readily computed from the time histories of angular velocities. Skull fracture in not a major concern in vehicular crashes^16^ due to the presence of airbags and thus linear acceleration based head injury criterion (HIC) was not considered.

## Methods

### Data

A supervised deep learning model takes in the inputs and the corresponding outputs and learns the mapping between the inputs and the outputs. For developing a deep learning model for predicting time history of angular velocities from crash videos, crash videos are required as inputs and the corresponding time history of angular velocities are required as outputs. FE based crash simulation data was utilized in this proof of concept study.

**Figure 1:**
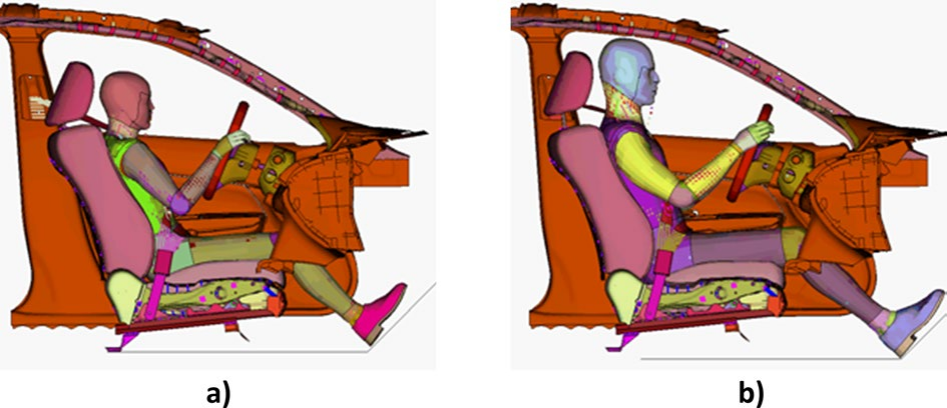
GHBMC human models in driving position (**a**) 5th female, (**b**) 50th male.

To generate the data, validated simplified Global Human Body Models Consortium (GHBMC) 50th percentile male^17,18^ and 5th percentile female^19,20^ FE human models were used in a variety of frontal crash simulations. These human models were positioned in the driver compartment (Fig. 1) that was extracted from the validated FE model of a 2014 Honda Accord^21^.

A validated generic seatbelt system with retractor, pretensioner and load limiter was included in the model along with validated frontal and side airbags^21^. In addition, steering column collapse was implemented and was included in these simulations. The roof rails, side door, B-pillar, and floor were deformable in the full FE model, but were made rigid in this study. The knee bolster and A-pillar were kept deformable. The human models were positioned in the driver compartment based on clearance measurements taken from physical crash tests (NHTSA test number 8035 for 50^th^ male, NHTSA test number 8380 for 5th female; https://www-nrd.nhtsa.dot.gov/database/veh/veh.htm). The crash pulse used for the simulations was taken from a physical crash test (NHTSA test number 9476) and is shown in Supplementary document Sect. 1.

These human models were evaluated in full frontal test condition, following which a design of experiments (DOE) study was conducted. For the DOE study, both crash-related parameters and restraint-related parameters were varied (Table 1). The crash related parameters were Delta-V and principal direction of force (PDOF). The restraint parameters were both seatbelt and airbag related. The parameters were varied over a wide range to generate a range of head motions including cases where the head hits the steering wheel.

**Table 1:** Parameters and their ranges.

Parameter	Range
*Crash-related parameters*	
Delta-V	25–45 mph
PDOF	− 30° (near side)–30° (far side)
*Restraint-related parameters*	
Frontal & side airbag mass flow rate	± 25%
Frontal & side airbag firing time	5–70 ms
Collapsible column breaking force	3000–10,000 N
Load limiter	1000–5000 N
Pretensioner limiting force	1000–3000 N
Friction between head and front/side airbag	0–3

The crash pulse for the same vehicle may be different for different PDOF, frontal overlap, and type and stiffness of the impacting surface. In addition, for the same PDOF, frontal overlap, and impacting surface, the crash pulse can vary for different vehicles of the same size (e.g., mid-size sedans). To keep the number of variables manageable for the DOE study, crash pulse shape was kept constant. Only crash pulse magnitude was scaled to achieve different Delta-Vs.

**Figure 2:**
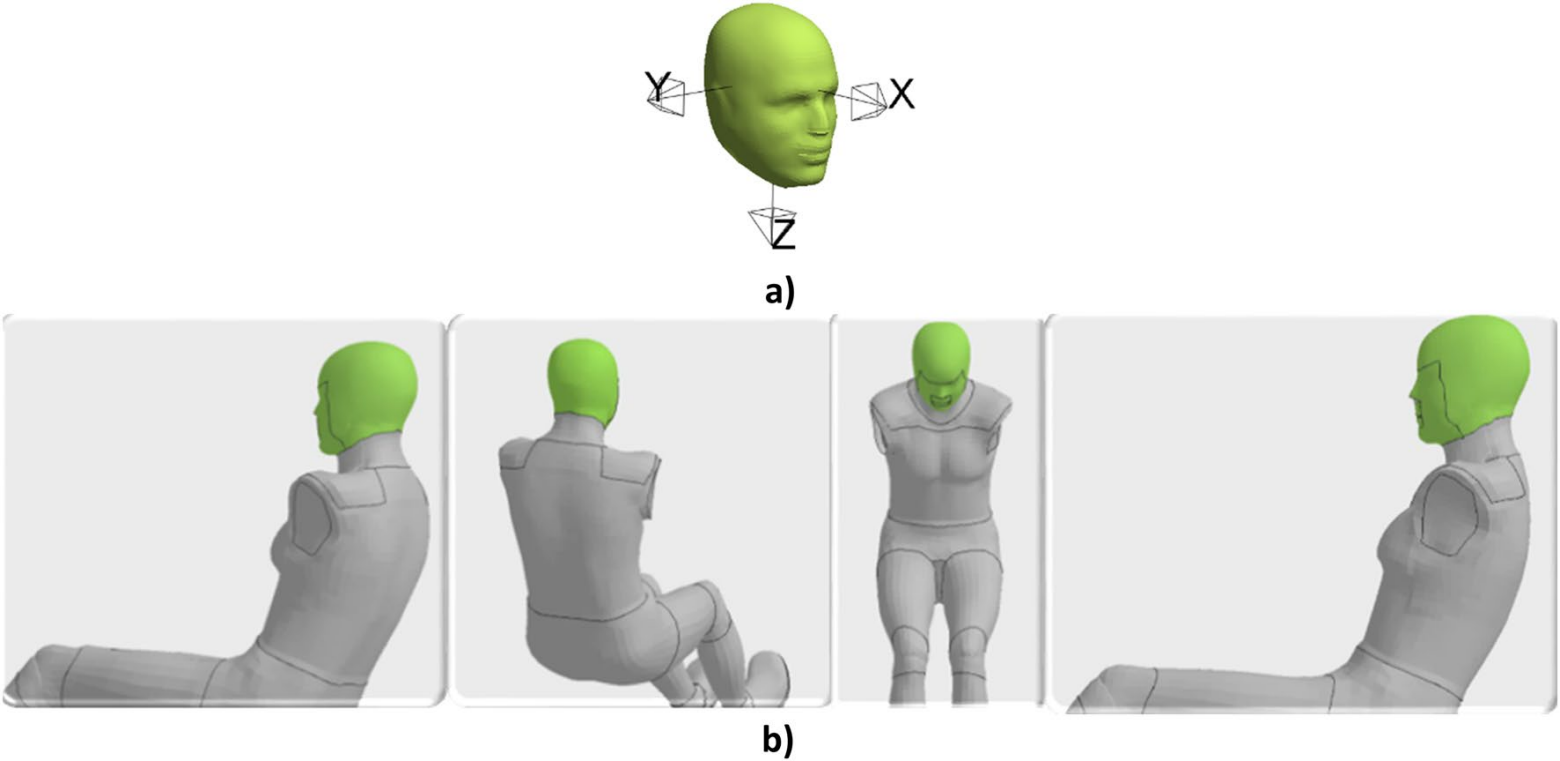
(**a**) Head rotational axes, and (**b**) Views used to generate videos: left isometric, back isometric, front isometric, and left.

A total of 1010 scenarios were simulated covering a wide range of crash conditions. Each crash scenario was simulated for a duration of 150 ms, which takes approximately 4.5 h on 28 processors. For each simulation, the time history of head angular velocities about the three head rotational axes (Fig. 2a) was computed and four crash videos with different views were generated (Fig. 2b).

The views chosen were similar to camera views available from NHTSA crash tests. Since the aim of the study was to predict the time history of head angular velocities from any view, each crash view was treated as a separate sample. Thus, we had a total of 4040 crash videos and their corresponding head angular velocity time histories (ω_x_, ω_y,_ ω_z_) about the three head rotational axes.

The crash videos were then used as inputs for the deep learning model and the corresponding angular velocity time histories were used as the “ground truth” outputs. For the purposes of this study, all crash videos were generated such that only the human model was visible. The vehicle structure and the airbags were removed from the videos to prevent any head occlusion.

Since videos are used as inputs to the deep learning model in the form of sequence of images, an additional input pre-processing step was carried out to convert the FE based crash videos to sequences of RGB images. Given the goal of this study was to predict the time histories of head angular velocities, the motion of the head was extracted as a sequence of RGB images over time from each FE crash video (Supplementary document Sect. 2). These sequences of images were then used as inputs to the deep learning model.

**Figure 3:**
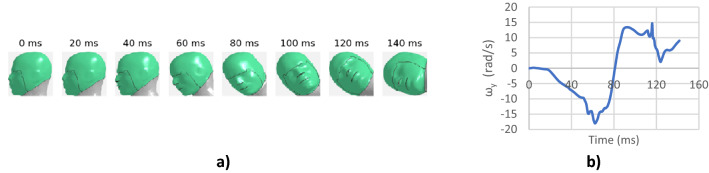
(**a**) Sample input, and (**b**) corresponding “ground truth” output for training.

The images were extracted every 2 ms from the 150 ms crash event and thus each sequence of images had a length of 76. The corresponding “ground truth” time histories of angular velocities (outputs or targets) were also sampled every 2 ms to match the corresponding sequence of images. This was done to support the deep learning architecture used in this study as described below in the deep learning model section. An example of the input and corresponding output for training the deep learning model is shown in Fig. 3. For visualization purpose, the input sequence of images in Fig. 3 is shown every 20 ms.

### Input data transformation

The input data (sequence of images) were RGB images with pixel values in the range from 0 to 255. Deep learning models train better and faster when input data is on the same scale. Thus, all the input sequences of images were normalized so that the pixel values were in the range from 0 to 1. Due to resource limitations, all images were resized to a height and width of 64 pixels and subsequently converted to grayscale such that each sequence of images had a shape of (76, 64, 64, 1), where number 76 stands for the number of images in a sequence, numbers 64 are for image size, and 1 stands for the number of channels (1 represents grayscale image).

### Data splitting

The entire dataset had 4040 samples. For developing the deep learning model, this dataset was split into three datasets: training, validation and test datasets. 74% the data was used for training, 13% of the data was used for validation and 13% of the data was used for testing. Data splitting was carried out using stratified sampling based on human model size and the crash view to ensure each of these (human model size and crash view) were equally represented in all three datasets (Supplementary document Sect. 3).

The training and validation datasets (87% of the data) were used for model development. The validation dataset was used for hyperparameters tuning and was a part of model development. The test dataset was not used in model development and was treated as an unseen dataset that was used to evaluate the final performance of the model.

### Deep learning model

The overall architecture for a deep learning model depends on the type of input data. The input data in this study is a sequence of images over time. Convolutional neural networks (CNN) can capture spatial dependency and are one of the most common types of neural networks used in computer vision to recognize objects and patterns in images. On the other hand, recurrent neural networks (RNN) can capture temporal dependency and are commonly used for sequential data processing. Thus, to process sequences of images in this study, a deep learning model that combines CNN^22^ and Long Short-Term Memory (LSTM)^23^ based RNN was used. The CNN-LSTM architecture uses CNN layers for feature extraction on input data combined with LSTMs to support sequence prediction.

Since the best architecture for our problem was not known at the start of model development, a lightweight baseline model (with fewer trainable parameters) was developed, which was later improved using hyperparameter tuning. For the CNN part of the baseline model, a Visual Geometry Group (VGG) style architecture^24^ was used, which consisted of a three-block network with two convolutional layers per block followed by a max pooling layer. Batch normalization^25^ and a rectified linear unit (ReLU) activation function^26^ were used after each convolutional layer. The baseline (initial) values selected for the number of convolutional filters for the three blocks were 16, 32 and 64 respectively. A global average pooling layer was added as the last layer of the CNN model to obtain the feature vector. Since each input sample is a sequence of images, the CNN part of the model was wrapped in a time distributed layer^27^ to get feature vector corresponding to the entire sequence. The time distributed wrapper helps apply the same CNN network to every temporal slice (image) of the input. The output from CNN was used as an input to the LSTM network. The LSTM network can be set up in multiple ways, the details of which are provided in Supplementary document Sect. 4. For the LSTM part of the baseline model, one LSTM layer with a hidden size of 128 was used. Since input sequence has a length of 76 and the goal is to predict the time history of angular velocity, the output was obtained at each recurring timestep from the LSTM layer. The output of the LSTM was then used as an input for a fully-connected layer with the ReLU activation function, followed by a dropout layer^28^ to control for overfitting. The output of the dropout layer was then fed to a fully-connected layer with a linear activation function to generate the final output, i.e. the predicted time history of angular velocity. Linear activation generates continuous numerical values and hence was used in the final output layer as angular velocity time history prediction was solved as a regression task.

The mean squared error (MSE) between the actual and predicted time history was used as the loss function for training the entire model. Adaptive moment estimation (Adam) optimizer^29^ was utilized for optimization. Since the ReLU activation was used in the network, He-Normal initializer^30^ was used to initialize the trainable weights of the model. The model was developed using Tensorflow v2.4^27^. The training was carried out on Google Colab using a single Tesla-P100 GPU. The model training time ranged from 1.5 to 2 h.

### Individual deep learning models and training

Training a single deep learning model to predict time history of all three components of angular velocity did not produce good results. Since the three components of angular velocity (ω_x_, ω_y,_ ω_z_) are independent of each other, three separate deep learning models were trained—one for each component of angular velocity ω_x_, ω_y,_ and ω_z_, which led to marked improvement in the results. The same training and validation inputs were used for training all three models. Only the “ground truth” targets were changed depending on the model. The baseline models for ω_x_, ω_y,_ and ω_z_ were trained with a learning rate of 0.0001 and with a batch size of 4 for a maximum of 80 epochs. Early stopping^27^ with a patience of 10 and model checkpointing^27^ callbacks were used to save the best model based on validation loss. Models often benefit from reducing the learning rate by a factor of 2–10 once learning stagnates. For this purpose, ReduceLROnPlateau^27^ callback was utilized. This callback monitors the validation loss and if no improvement is seen for 5 epochs, the learning rate is reduced.

The hyperparameter values chosen for the CNN, LSTM, and the extended part of the baseline model were selected at random and did not necessarily correspond to the best architecture for the problem. To improve the models, hyperparameter tuning (Supplementary document Sect. 5) was carried out to find the set of hyperparameter values that give the best results for our problem.

Because of resource limitations, hyperparameter tuning was only performed for the ω_x_ model to find the best set of hyperparameters. This set of hyperparameters was then used to train the final deep learning models for all three components of angular velocity.

### Combined model

**Figure 4:**
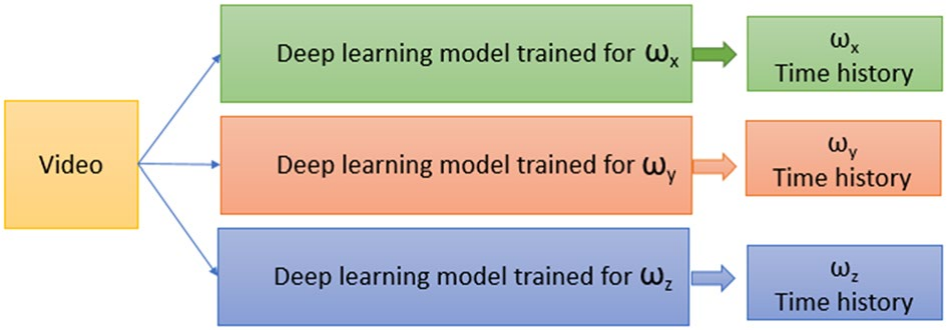
Combined deep learning model.

The three individually trained models for ω_x_, ω_y,_ and ω_z_ were combined into a single deep learning model as shown in Fig. 4. To predict the time history of the three components of angular velocity from a video input of any view, the video (preprocessed as sequence of images) is passed into the combined model. It is then propagated (forward pass) through the individually trained networks that output the time history of the three components of angular velocity ω_x_, ω_y,_ and ω_z._

### Model evaluation

#### Individual model evaluation

The three individually trained deep learning models for ω_x_, ω_y,_ and ω_z_ were evaluated on the test dataset to see how well they generalize on unseen data. The actual and predicted time histories for cases from the test dataset were compared quantitatively using CORA^31^. While time histories of angular velocities are important to assess overall head kinematics, for computing brain injury metrics peak values are usually used. For example, brain injury criterion (BrIC)^4^ is computed using absolute peaks of ω_x_, ω_y,_ and ω_z_ (Eq. (1)).

1 $${\text{BrIC}} = \sqrt {\left( {\frac{{\omega_{xm} }}{66.25}} \right)^{2} + \left( {\frac{{\omega_{ym} }}{56.45}} \right)^{2} + \left( {\frac{{\omega_{zm} }}{42.87}} \right)^{2} }$$

To evaluate prediction of the peak angular velocity, correlation coefficient between the actual and predicted peaks was computed for all three models using the test dataset.

#### Frame rate evaluation

**Figure 5:**
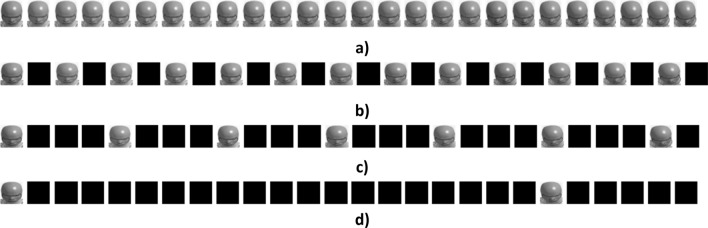
Input sequence of Images for (**a**) 500 fps, (**b**) 250 fps, (**c**) 125 fps, and (**d**) 25 fps. Image sequences shown for first 50 ms only for visualization purposes.

The individual models were trained on a sequence of images captured every 2 ms, i.e. 500 frames/second (fps) videos (Fig. 5a). Evaluation was carried out to determine the influence of frame rate on both time history and peak predictions. Three different frame rates were evaluated i.e. 250 fps, 125 fps and real time video at 25 fps. For this evaluation, the “ground truth” angular velocities sampled every 2 ms were kept the same, but the input sequence of images were changed. For 250 fps, images sampled every 4 ms were kept in the sequence of images while others were converted to a black image (Fig. 5b). Similarly, only images sampled at 8 ms and 40 ms were kept for 125 fps (Fig. 5c) and 25 fps (Fig. 5d) while the rest were converted to black images. This procedure was followed to support the deep learning architecture described above.

Models were trained for ω_x_, ω_y,_ and ω_z_ for each frame rate using the same hyperparameters as in the 500 fps study. These models were then evaluated on the test dataset by quantitatively comparing the actual and predicted time histories using CORA for the same cases as the 500 fps study. In addition, correlation coefficients between the actual and predicted peaks were evaluated.

#### Combined model evaluation

The combined model was evaluated for a few cases from the test dataset. For these cases, the actual and predicted time histories were compared using CORA. These actual and predicted time histories were also used to simulate the SIMon head model^15^ to compare the actual and predicted brain strains. In addition, the actual and predicted BrIC values were compared.

#### Camera view performance evaluation

Since four different views were used in this study to train the models, evaluation was conducted using the test dataset to determine the performance of each view. For each view, average CORA scores were computed for all three components of angular velocity (ω_x_, ω_y,_ and ω_z_). Correlation coefficients between the actual and predicted peaks for the three components of angular velocity were computed for each view as well. Both the average CORA scores and correlation coefficients were used to make the performance determination.

#### Additional crash pulse evaluation

**Figure 6:**
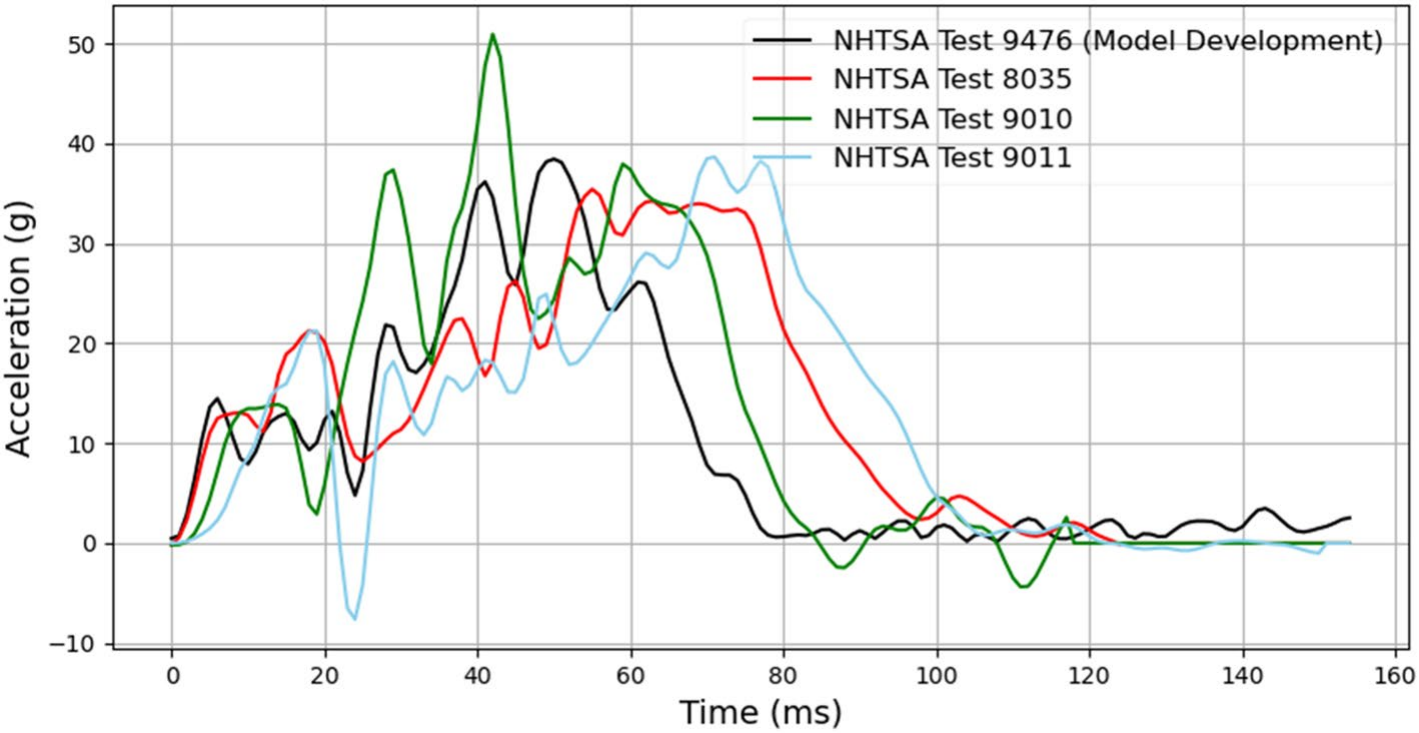
Crash pulses from NHTSA crash tests.

The models for ω_x_, ω_y,_ and ω_z_ were trained using a single crash pulse (Supplementary document Sect. 1), which was taken from NHTSA test 9476 (2015 Chevrolet Malibu in a frontal oblique offset test). The crash pulse magnitude was changed but shape was kept the same. To test the robustness, the combined model was further evaluated using three additional crash pulses (Fig. 6). These crash pulses were taken from the NHTSA test numbers 8035 (2013 Honda Accord in a frontal Impact test), 9010 (2015 Ford Escape in a frontal Impact test), and 9011 (2015 Dodge Challenger in a frontal Impact test). Frontal impacts were simulated with these additional crash pulses using the 50th male GHBMC model. Videos were then generated for these simulations and the combined deep learning model time history predictions were compared with the actual time histories of head angular velocities.

## Results

### Final model architecture

**Figure 7:**
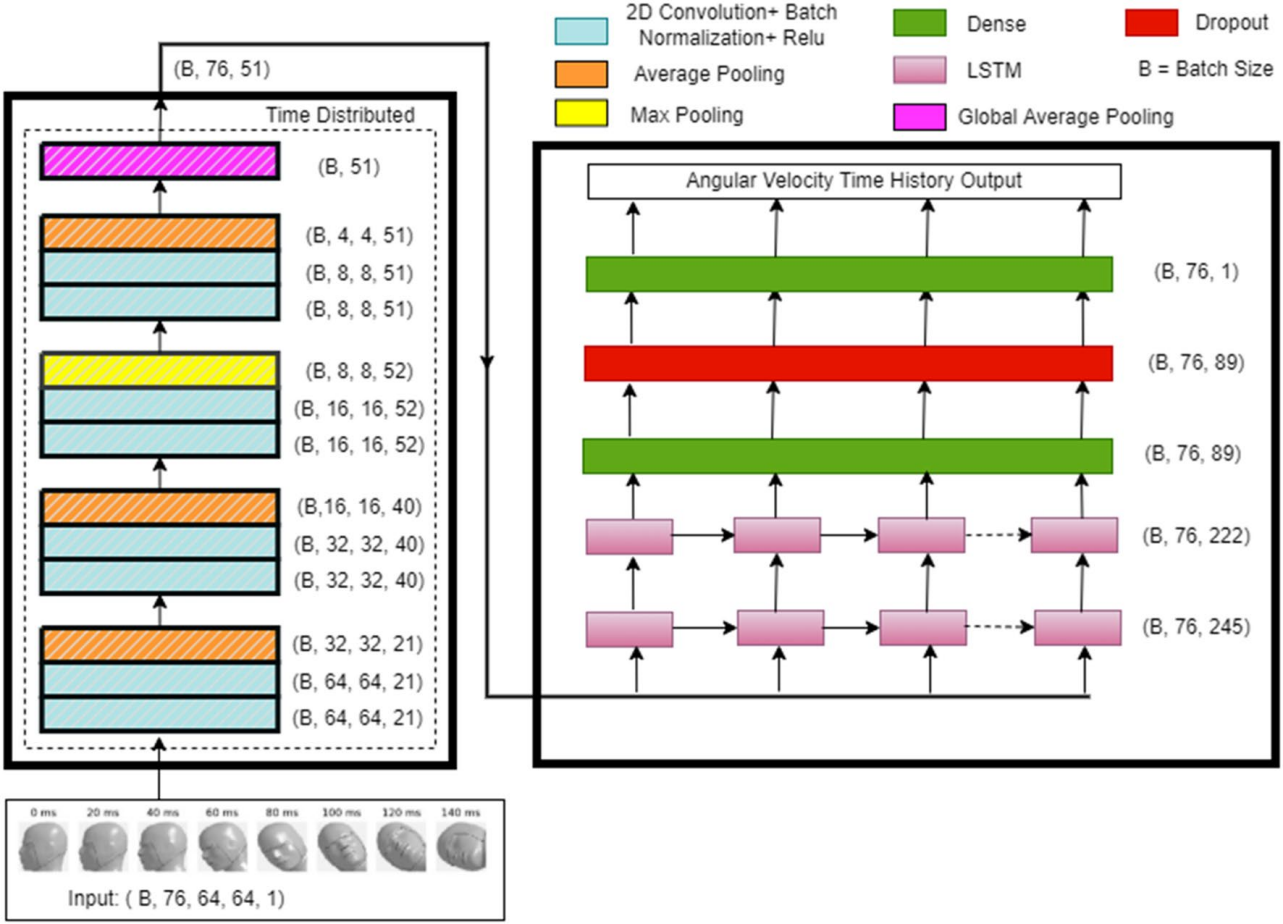
Final model architecture.

Figure 7 shows the final model architecture with tuned hyperparameters (Supplementary document Sect. 5, Supplementary Table S2). The data shapes shown in Fig. 7 are the output shapes from each layer. This model has approximately 845,000 trainable parameters.

### Model evaluation

#### Individual model evaluation

**Figure 8:**
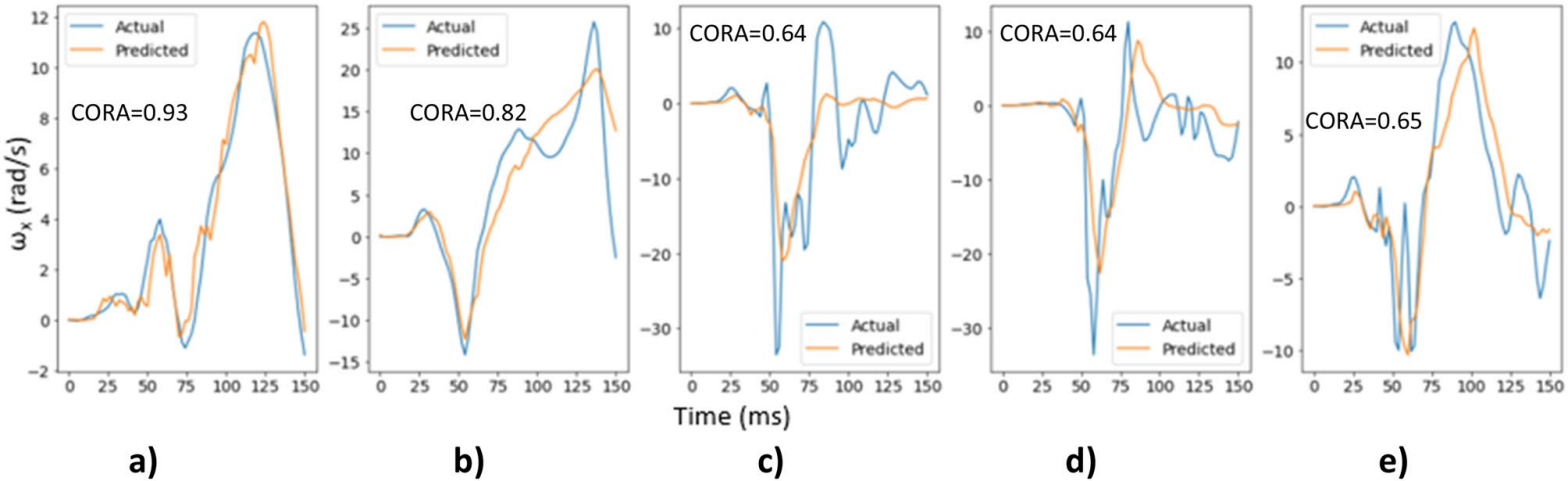
Actual and predicted time histories for ω_x_ for 5 random cases from the test dataset.

The final deep learning models (with tuned hyperparameters) for ω_x_, ω_y,_ and ω_z_ were assessed on the test dataset to evaluate how well they generalize on unseen data. Figure 8 shows the actual and predicted time histories for ω_x_ for 5 randomly selected cases from the test dataset along with their respective CORA score. It can be observed that the ω_x_ deep learning model is able to predict the time histories reasonably well for these cases.

**Figure 9:**
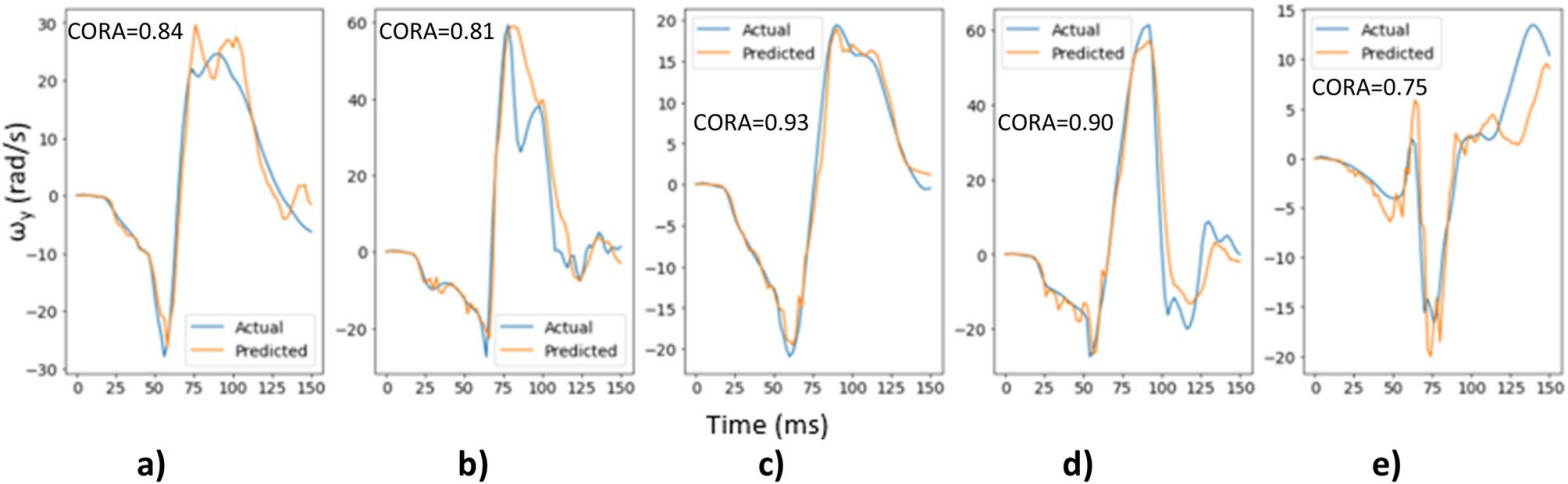
Actual and predicted time histories for ω_y_ for 5 random cases from the test dataset.

Figure 9 shows the actual and predicted time histories for ω_y_ for 5 randomly selected cases from the test dataset along with their respective CORA score, which demonstrates better prediction of the time histories for ω_y_ component than those for ω_x_.

**Figure 10:**
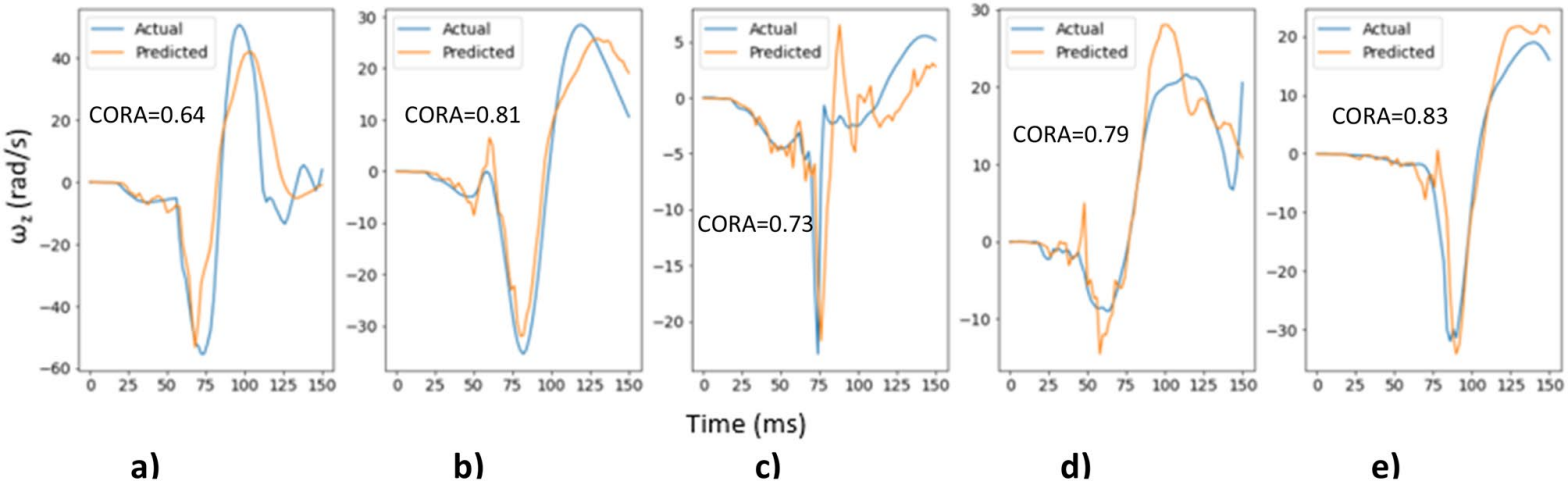
Actual and predicted time histories for ω_z_ for 5 random cases from the test dataset.

Similarly, Fig. 10 shows the actual and predicted time histories for ω_z_ for 5 randomly selected cases from the test dataset along with their respective CORA score demonstrating a reasonable match between the actual and predicted time histories. Additionally, Fig. 10 shows different types of head impacts: (1) head contacts the airbag (Fig. 10a,b,d,e), and (2) head contacts the steering wheel (Fig. 10c). The model is able to predict the time history for these different head impacts demonstrating that the trained deep learning models are capable of learning important features that distinguish between different types of head impacts.

**Figure 11:**
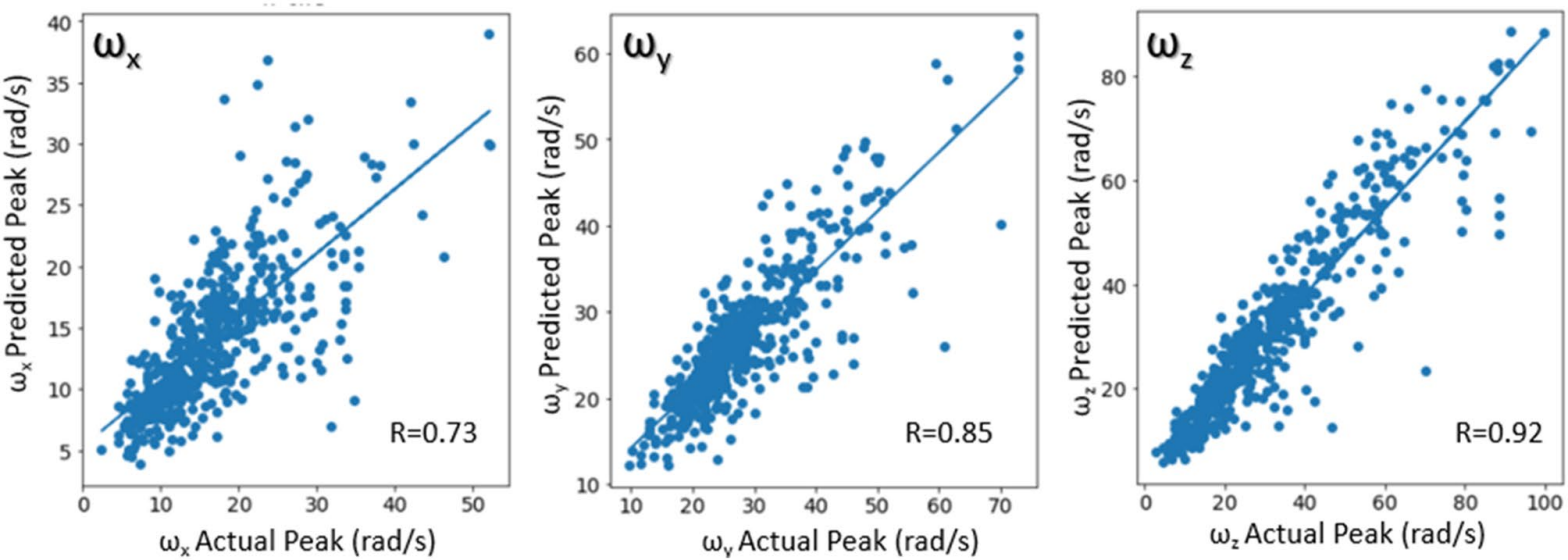
Correlation plots for ω_x_, ω_y,_ and ω_z_.

The peak angular velocities were evaluated quantitatively (Fig. 11) with correlation plots using only the unseen test dataset. Correlation coefficients were 0.73 for ω_x_, 0.85 for ω_y_ and 0.92 for ω_z_.

#### Frame rate evaluation

Figures 8, 9, 10 and 11 above show the results obtained with 500 fps videos. The time history results for the other three frame rates (250 fps, 125 fps and 25 fps) are shown in Supplementary document Sect. 6. Table 2 shows the correlation coefficients across the different frame rates. It can be observed that at 250 fps, the CORA scores and correlation coefficients drop slightly compared to 500 fps results, but the results are affected more at lower frame rates with poor predictions and correlations at 25 fps (real time videos). This is expected as crash events last ~ 150 ms and high speed videos are necessary to capture head kinematics.

**Table 2:** Correlation coefficients for different frame rates.

Frame rate	Correlation coefficient between actual and predicted peaks		
	Angular velocity-X	Angular velocity-Y	Angular velocity-Z
500 fps	0.73	0.85	0.92
250 fps	0.68	0.80	0.91
125 fps	0.64	0.70	0.83
25 fps	0.00	0.34	0.49

#### Combined model evaluation

**Figure 12:**
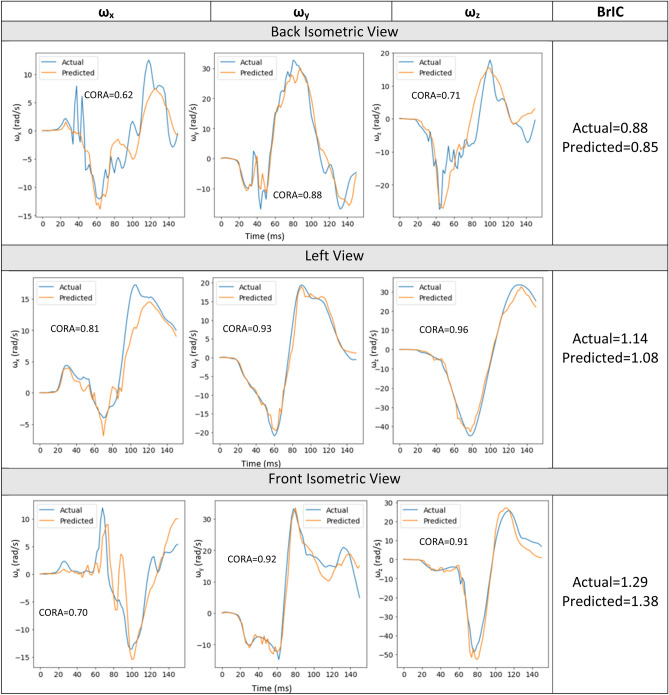
Combined model results.

The combined model performance was also assessed on the test dataset. Three random videos with different views (back isometric view, front isometric view and left view) were selected from the test dataset and the 3D angular kinematics predicted by the combined model were compared with the actual data. The actual and predicted time history results for these three videos along with their respective CORA score are shown in Fig. 12. The actual and predicted BrIC values are also included in Fig. 12.

**Figure 13:**
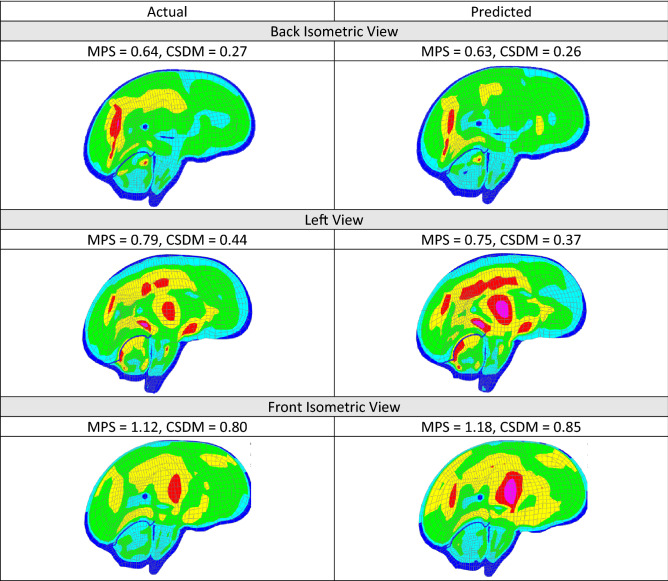
Brain strain comparison.

The actual and predicted time histories from the three cases (Fig. 12) were applied to the SIMon head model to compare the brain strains. Similar actual and predicted brain strain patterns were observed for the three cases (Fig. 13). MPS and CSDM (0.25) values were computed and are also given in Fig. 13.

#### Camera view performance evaluation

**Figure 14:**
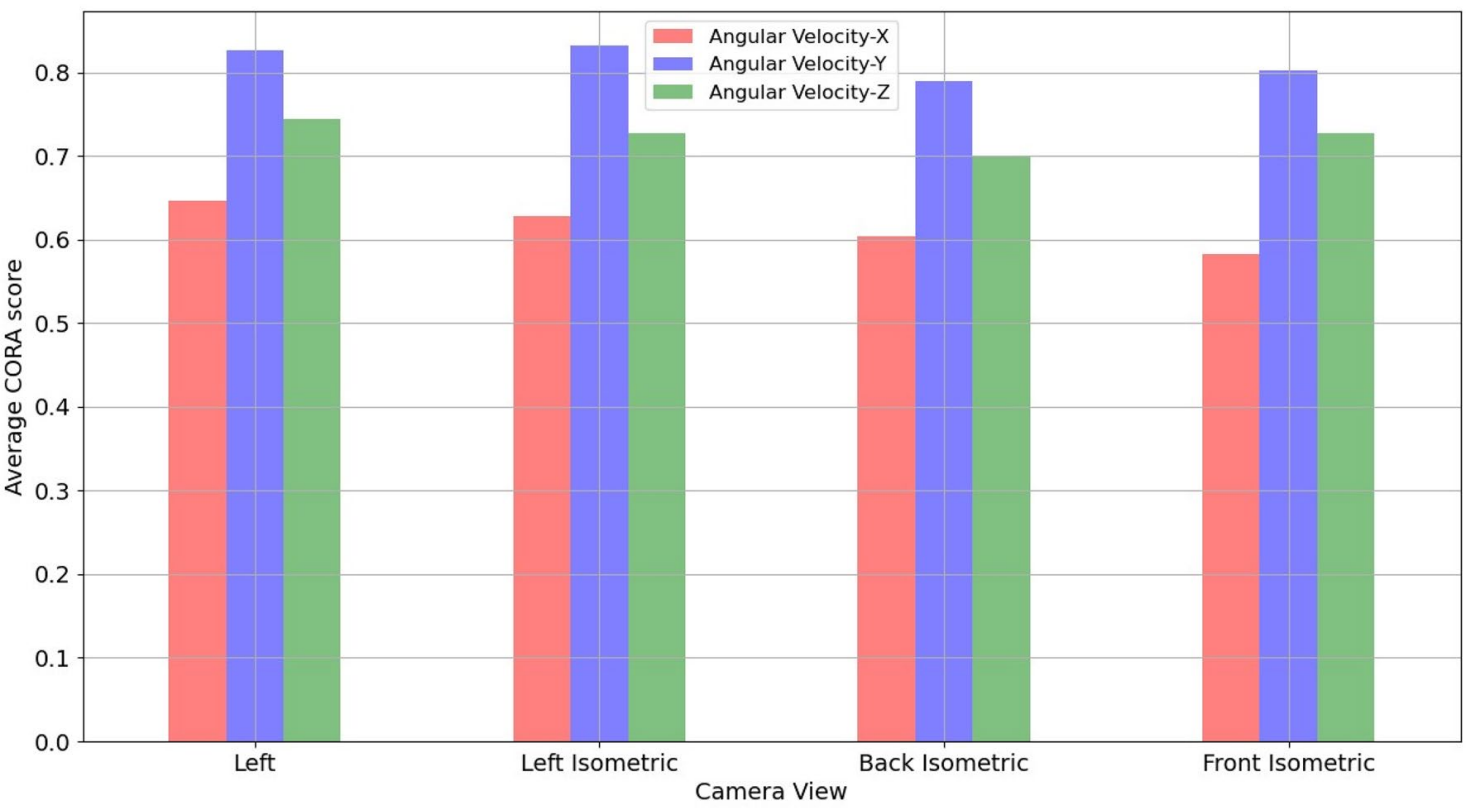
Average CORA scores for each camera view.

The four camera views used in this study were evaluated on test dataset to determine the view with best predictions. Figure 14 shows the average CORA scores for ω_x_, ω_y,_ and ω_z_ for each view. An overall average CORA score (average of averages) across the three angular velocities was computed for all four views. The left and left isometric views showed the best overall average CORA score (~ 0.73) followed by back isometric and front isometric views (~ 0.70).

Table 3 shows the correlation coefficients between actual and predicted angular velocity peaks for the four views. Average correlation coefficient across the three angular velocities was computed for all four views. Based on this average score, the left isometric view showed the best results (0.88), followed by left view (0.84), back isometric view (0.82) and finally front isometric view (0.79).

**Table 3:** Correlation coefficients for different camera views.

Camera view	Correlation coefficient between actual and predicted peaks			Average correlation coefficient
	Angular velocity-X	Angular velocity-Y	Angular velocity-Z	
Left	0.71	0.89	0.93	0.84
Back isometric	0.71	0.84	0.90	0.82
Left isometric	0.79	0.91	0.93	0.88
Front isometric	0.70	0.75	0.93	0.79

Based on CORA scores (which considers various aspects of the time history signal, such as size, shape, and phase^31^) and correlation coefficients (based on peaks in this study and important for injury metric computation), left isometric view demonstrates the best performance.

#### Additional crash pulse evaluation

**Figure 15:**
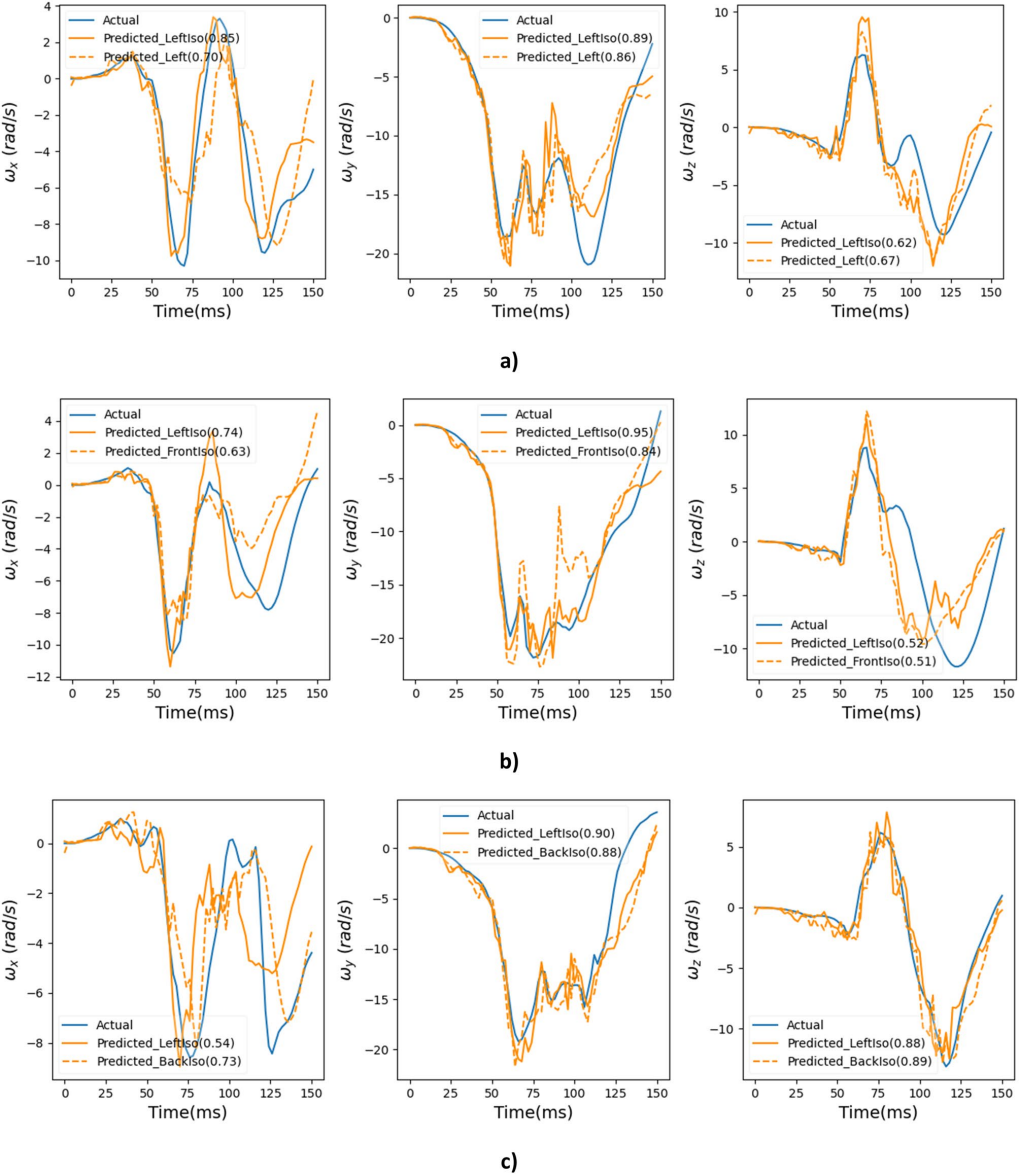
Angular velocity time history comparison: (**a**) left and left isometric views (Crash pulse—NHTSA test 8035), (**b**) left isometric and front isometric views (Crash pulse—NHTSA test 9010), and (**c**) left isometric and back isometric views (Crash pulse—NHTSA test 9011).

Comparison between actual and predicted angular velocity time histories for simulated crash videos based on crash pulses taken from NHTSA crash tests 8035, 9010 and 9011 is shown in Fig. 15. Results are presented from two different views with their respective CORA score shown in parentheses. For all cases, results from left isometric view are presented (as it showed the best performance (Fig. 14 and Table 3)) along with a different second view. The model shows promising predictions for all three angular velocities on these out of sample (unseen) crash videos.

## Discussion

The objective of this study was to evaluate the feasibility of deep learning methodology for predicting head angular kinematics directly from simulated crash videos. The methodology was demonstrated by developing a proof of concept model using FE data for predicting angular velocity time history.

### Main findings

Deep learning models are function approximators that approximate the unknown underlying mapping function from inputs to outputs. The results of this study show that deep learning models for ω_x_, ω_y,_ and ω_z_ are capable of effectively capturing important features from the input sequence of images and mapping them to the output angular velocities, thus demonstrating the feasibility of the deep learning approach for predicting angular head kinematics.

The deep learning models showed promising results when evaluated on the test dataset (Figs. 8, 9, 10, 11 and 12), and additional crash pulse dataset (Fig. 15) demonstrating their ability to generalize well on unseen data. The SIMon head model simulations with three different actual and predicted angular velocity time histories showed comparable MPS, CSDM, and brain strain patterns (Fig. 13). The actual and predicted BrIC values were also similar for these cases (Fig. 12).

Deep learning models have been shown to work well on unstructured data, such as images, videos, text etc. Depending on the task, deep learning models can have a large number of trainable parameters (tens of millions) and are thus trained on large datasets to avoid overfitting. For example, image classification models are trained using the popular ImageNet dataset that has millions of images. Biomechanical datasets are very limited for example, Wu et al.^7^ used a dataset of size 3069 to develop a deep learning model for efficient estimation of regional brain strains and Zhan et al.^8^ used data from 1803 head impacts for developing their deep learning model for estimation of entire brain deformation in concussion. In this study, the data size was 4040, which was generated using 1010 FE simulations. Since dataset size is small, in addition to using regularization techniques like dropout to avoid overfitting, the number of trainable parameters were also kept under check. The final deep learning model had approximately ~ 845 K trainable parameters. Despite using this rather small dataset, the deep learning models for ω_x_, ω_y,_ and ω_z_ were capable of predicting the time histories and their respective peaks well (Figs. 8, 9, 10 and 11). This demonstrates that deep learning methodology may be feasible with small datasets, while more data could possibly lead to better predictive models.

The 4040 data points used in this study come from 1010 different FE simulations. The four views generated per simulation were used as separate inputs that were not combined into one input. Combining the four views into a single sample would require the availability of those four views for every prediction and that might become a bottleneck if certain views are not available. The methodology used herein—treating each view as a separate input provides the advantage of predicting kinematics from any available view that model is trained on. In addition, the deep learning methodology does not need multiple views to get 3D kinematics and provides the advantage of obtaining 3D kinematics from any 2D view as shown in Fig. 12.

We used a deep learning approach to predict head angular kinematics from crash events that last ~ 150 ms. To predict angular velocity time history from events that have similar duration, high speed cameras are preferred for better accuracy (Figs. 8, 9 and 10, Table 2, Supplementary document Sect. 6). For short duration dynamic events, such as a car crash, it is difficult to predict the time history using frames of images spaced out by 40 ms (real time videos at 25 fps) as compared to predicting the time history using frames of images that are 2 ms apart (high speed camera at 500 fps). The higher the frame rate, the better the time history predictions for the short duration events. This may also be due to insufficient sample size when sampling a short event with lower frame rate. The longer events (greater than 150 ms) at low frame rates were not evaluated in this study. The results (Figs. 8, 9 and 10, Table 2, Supplementary document Sect. 6) show that high speed cameras processing at or above 250 fps provide reasonable results for 150 ms events.

The x-component of the angular velocity vector (ω_x_) showed lower CORA scores (Fig. 14) and had more discrepancy in predicting peak values (Fig. 11) when compared to ω_y,_ and ω_z_. The reason for this discrepancy may be due to the views (left, left isometric, front isometric and back isometric) selected for training the models. These views may be more conducive for learning important features related to prediction of ω_y,_ and ω_z_, but not so much for ω_x_. Based on CORA scores and correlation coefficients (Fig. 14, Table 3), the best camera view for ω_x,_ and ω_y_ was the left isometric view. For ω_z,_ left, left isometric, and front isometric views provided equally good results. Overall, left isometric camera view demonstrated best performance.

The deep learning methodology shows promising results even with low resolution (64,64) grayscale images and limited hyperparameter tuning. Given enough resources, better models may be developed for real world applications by using this methodology with higher resolution RGB images, exploring a wider range of hyperparameters and using more advanced CNN architectures like Residual networks^32^, EfficientNets^33^ etc. that may help the networks learn better features from the input sequence of images, thus further improving predictions.

### Applicability to NHTSA crash tests

The methodology in this study demonstrated good results in a controlled FE environment where the head characteristics are the same for all data samples, similar camera angles are available, and unobstructed view is available for the duration of the crash event. Thus, this methodology holds promise for real-world controlled environment applications, for example testing environments like NHTSA crash tests where anthropomorphic test devices (ATDs) have similar head characteristics, fixed camera angles are available and unobstructed view is available from a back camera. Predictive models, once developed using this deep learning methodology, from NHTSA crash test data may be used to obtain head 3D rotational kinematics from the crash test videos involving 5th percentile ATD, which have limited head instrumentation and currently don’t have sensors to output rotational head kinematics.

### Comparison with other related work

We used a deep learning architecture that combines CNN and LSTM to capture spatial and temporal dependency respectively. A sequence of 2D head images served as an input while the time history of head angular velocity was the model output. Various deep learning architectures have been used in brain injury biomechanics field. For example, Wu et al.^7^ used a CNN architecture to predict regional brain strain by using 2D images of the head angular kinematics profiles as input. Similarly, Ghazi et al.^9^ applied CNN model to estimate element-wise strain in the brain by using 2D images of rotational velocity and acceleration 2D profiles. In both studies, the input was a single image (not a sequence of images) and the output was not the time history profile and thus they did not require use of LSTMs. Bourdet et al.^10^ used a U-Net style architecture^34^ with 1D convolutions to output the time history of the maximum Von Mises stress in the entire brain using time history of three linear accelerations and three angular velocities as an input. U-Net architectures are fully convolutional networks that were developed for biomedical image segmentation. Since U-Net architecture generates output with the same spatial dimension as the input because of its encoder-decoder structure, it was not feasible for our 2D image dataset as the goal of our study was to extract feature vectors from the sequence of 2D images for time history prediction.

### Limitations

Deep learning methodology has many advantages, but it is not without limitations. For example, the proof of concept model developed in this study cannot be directly applied to real world scenarios (for example, NHTSA physical crash tests with ATDs) because the features learned by the model are from GHBMC human head model and may not be directly applicable to other images, such as other human heads/models or ATD head. However, the methodology described herein may be extended to build predictive models for such real-world scenarios. We developed the deep learning models from FE crash videos with an unobstructed view of the head. Head occlusion can lead to head features that may not be correctly identified, which would give inaccurate prediction of angular velocities. For real-world data, the head may get obstructed at some point in the event in some camera views. Thus, it is recommended to develop predictive models for real world applications using data from camera angles in which the head remains visible (or partially visible) throughout the event. In addition, deep learning models work well with the type of data they are trained on (i.e. deep learning models may not be well suited for extrapolation). For example, models trained on just the front views cannot be expected to make correct predictions based on back view videos. Thus, when making predictions, videos from camera angles similar to those used in the model training may be necessary. This kind of limitation has also been pointed out by Bourdet et al.^10^ and Ghazi et al.^9^ for their models.

### Advantages of deep learning approach

Training deep learning models require high end machines with GPUs. However, once trained such models can be used to make angular velocity predictions in a few milliseconds from a given crash video. The inference (prediction) time for the combined model in this study was 117 ms on a GPU. The advantage of deep learning approach is that with the availability of new data, the training dataset may be appended for retraining, thus allowing for iterative improvement of the model.

While we developed deep learning models for predicting angular velocity time histories, the approach is not limited to angular velocities. It may be used for predicting other kinematic parameters, such as linear accelerations and linear acceleration based head injury criterion (HIC) etc.

Predicting head kinematics using deep learning methodology provides an alternative method of computing head kinematics and may be deployed in situations where the sensor data is either not available or insufficient to determine head angular kinematics (assuming that videos of such events are available).

## Conclusions

Proof-of-concept deep learning models developed in this study showed promising results for predicting angular velocity time histories, thus demonstrating the feasibility of deep learning methodology.

### Future work

Future work involves extending this deep learning methodology to NHTSA crash tests for predicting ATD head angular kinematics.

## Supplementary Information

Supplementary Information.
