# Supplementary material for: Deep learning methodology for predicting time history of head angular kinematics from simulated crash videos
Source: Sci Rep. 2022 Apr 20;12:6526. doi: 10.1038/s41598-022-10480-w (PMC9021239; doi:10.1038/s41598-022-10480-w)
Supplement: Supplementary file 1 — Supplementary Information. [file 41598_2022_10480_MOESM1_ESM.docx]

**Supplementary Information**

**Deep Learning Methodology for Predicting Time History of Head Angular Kinematics from Simulated Crash Videos**

**Vikas Hasija**^1^ **and** **Erik G. Takhounts**^2^

^1^Bowhead (Systems & Technology), Washington, DC, USA

^2^National Highway Traffic Safety Administration (NHTSA), Washington, DC, USA

*References for supplementary information are in this file.*

**Section 1: Crash Pulse**

Supplementary Figure S1 shows the crash pulse used for the simulations.

Delta-V=33 mph

**Supplementary Figure S1**. Crash pulse.

**Section 2: RGB image extraction from crash videos**

To extract the motion of the head over time as a sequence of RGB images, the head needs to be detected in each frame of the crash video. A head detection algorithm may be employed for this purpose. The use of FE based crash videos in our study offered an additional advantage in utilization of a fast and accurate computer vision-based color mask as a head detection algorithm. In all FE crash videos, the head of the human model was colored green and the rest of the body was kept gray such that the head could be easily detected in each frame of the video with a bounding box using Contours in OpenCV^1^ (Supplementary Figure S2). Once detected, the head image inside the bounding box was extracted from each frame of the video as a RGB image (Supplementary Figure S2) to obtain a sequence of head images over time.

| 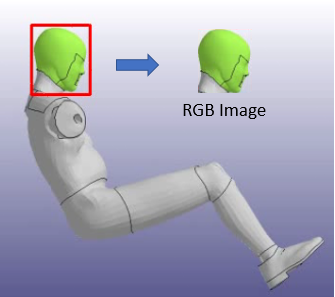 |
| --- |
| **Supplementary Figure S2.** Shown is a single frame of a crash video with the red bounding box generated by the Contours technique in OpenCV used for extraction of RGB image of the head. |

The Contours based detection technique gave zero false positives and generated a complete sequence without missing any frames. However, it only works if the user has a full control over all color aspects of the videos, which was the case in our study. For a “real world condition” (not based on the simulations), a head detection model with a tracking algorithm, such as Kalman filter, may be more appropriate.

**Section 3: Data splitting**

The count plot (Supplementary Figure S3a) shows the distribution of data for each human model size and for each view for the full dataset. The count plots for training, validation and test datasets are shown in Supplementary Figure S3b.

| 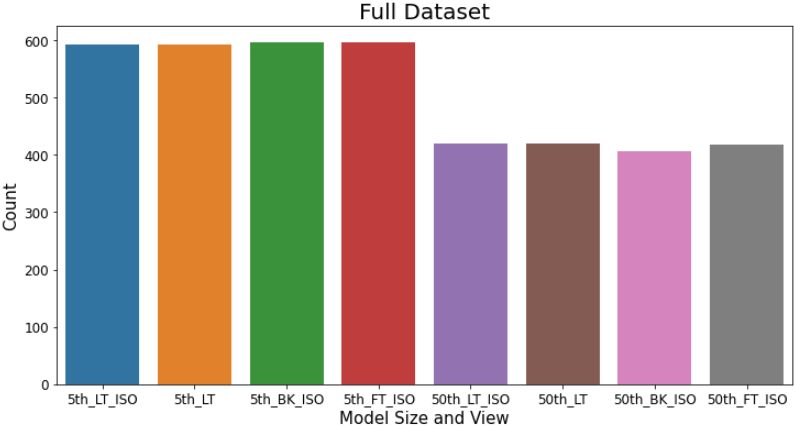  *5^th^: 5^th^ Female*  *50^th^: 50^th^ Male*  *LT_ISO: Left Isometric view*  *LT: Left view*  *BK_ISO: Back Isometric view*  *FT_ISO: Front isometric view*  **a)** |
| --- |
| 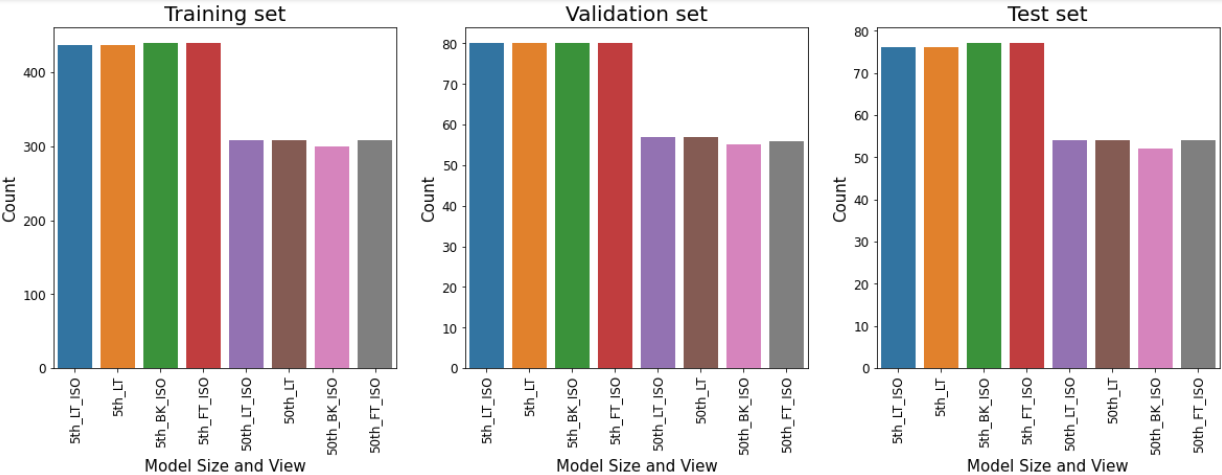  **b)** |
| **Supplementary Figure S3.** Count plot for a) Full dataset, and b) Training, Validation and Test datasets. |

**Section 4: LSTM set up**

LSTM network can be set up in two different ways for this problem, i.e. process the output sequence from the time distributed CNN using LSTM: a) use only the LSTM output from the last time step to generate the time history output (Supplementary Figure S4a), or b) use LSTM output at each time step to generate the time history output (Supplementary Figure S4b). The first option does not require the input image sequence and “ground truth” outputs to be sampled at the same frequency, but it is required for the second option. Generating the entire angular velocity time history using just the final LSTM output (option a)) did not show good results in our preliminary testing when compared to option b), which was eventually selected for this study. To support this architecture, the “ground truth” time histories of angular velocities were sampled every 2 ms to match the corresponding sequence of images.

| 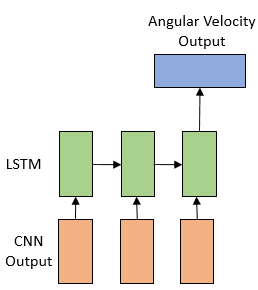 | 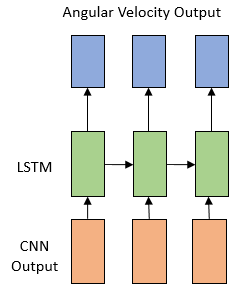 |
| --- | --- |
| **a)** | **b)** |
| **Supplementary Figure S4.** LSTM network architecture options. | |

**Section 5: Hyperparameter tuning**

Supplementary Table S1 shows the hyperparameters that were varied along with their corresponding range. Validation loss was tracked to find the best set of hyperparameters. Keras-Tuner^2^ was used for hyperparameter tuning using Bayesian Optimization^3^.

| **Hyperparameters** | **Baseline value** | **Range explored** |
| --- | --- | --- |
| **CNN Based** | | |
| Number of VGG blocks | 3 | 1 – 5 |
| Number of convolutional filters per block | 16,32,64 | 16 - 64 |
| Pooling type for each block | max | max, average |
| **LSTM Based** | | |
| Number of LSTM layers | 1 | 1-2 |
| Number of LSTM units per layer | 128 | 64 - 256 |
| **Others** | | |
| Number of units for fully-connected layer | 80 | 64 - 128 |
| Dropout rate | 0.5 | 0.0 – 0.5 |
| Learning rate | 1e-4 | 1e-4 – 1e-2 |
| **Supplementary Table S1**. Hyperparameters. | | |

Supplementary Table S2 shows the optimized hyperparameters obtained after hyperparameter tuning.

| **Hyperparameters** | **Optimized value** |
| --- | --- |
| **CNN Based** | |
| Number of VGG blocks | 4 |
| Number of convolutional filters per block | 21, 40, 52, 51 |
| Pooling type for each block | average, average, max, average |
| **LSTM Based** | |
| Number of LSTM layers | 2 |
| Number of LSTM units per layer | 245, 222 |
| **Others** | |
| Number of units for fully-connected layer | 89 |
| Dropout rate | 0.32 |
| Learning rate | 0.00018 |
| **Supplementary Table S2.** Optimized Hyperparameters. | |

**Section 6: Frame rate evaluation**

The time history results for frame rates of 250 fps, 125 fps and 25 fps are shown in Supplementary Figures S5, S6 and S7 respectively.

| 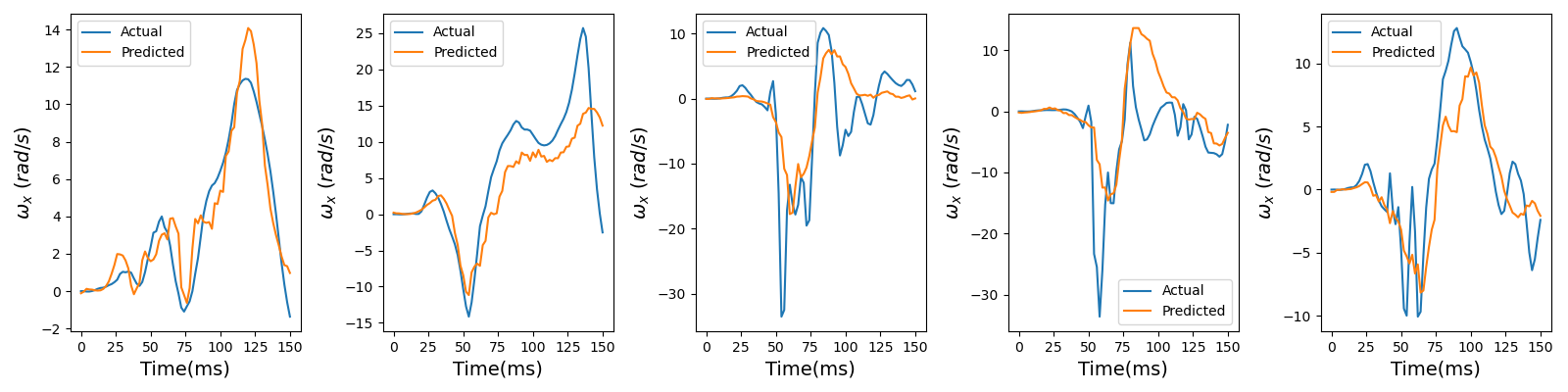  CORA=0.84  CORA=0.60  CORA=0.70  CORA=0.50  CORA=0.60 |
| --- |
| **a)** |
| 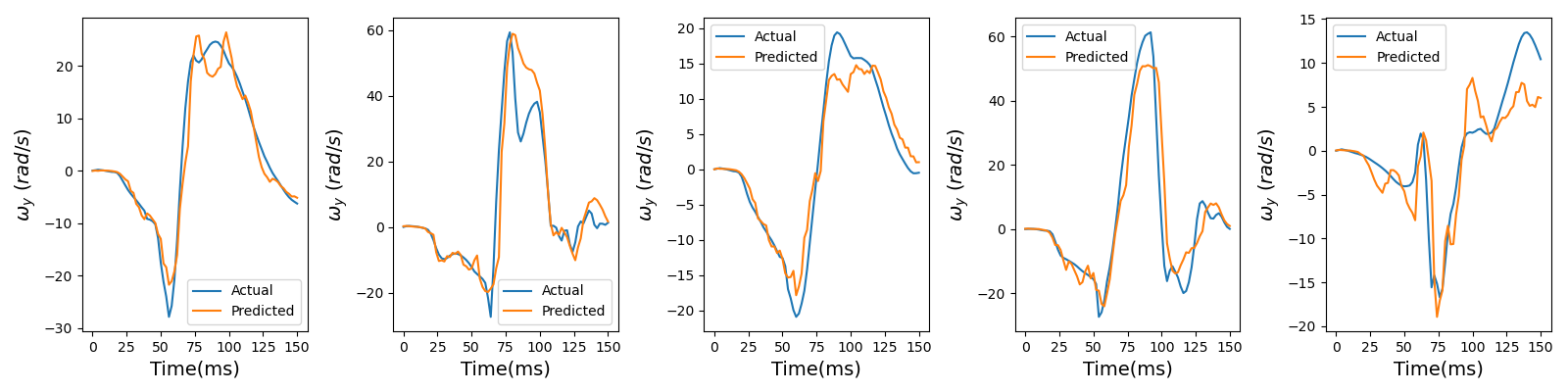  CORA=0.72  CORA=0.84  CORA=0.81  CORA=0.85  CORA=0.80 |
| **b)** |
| 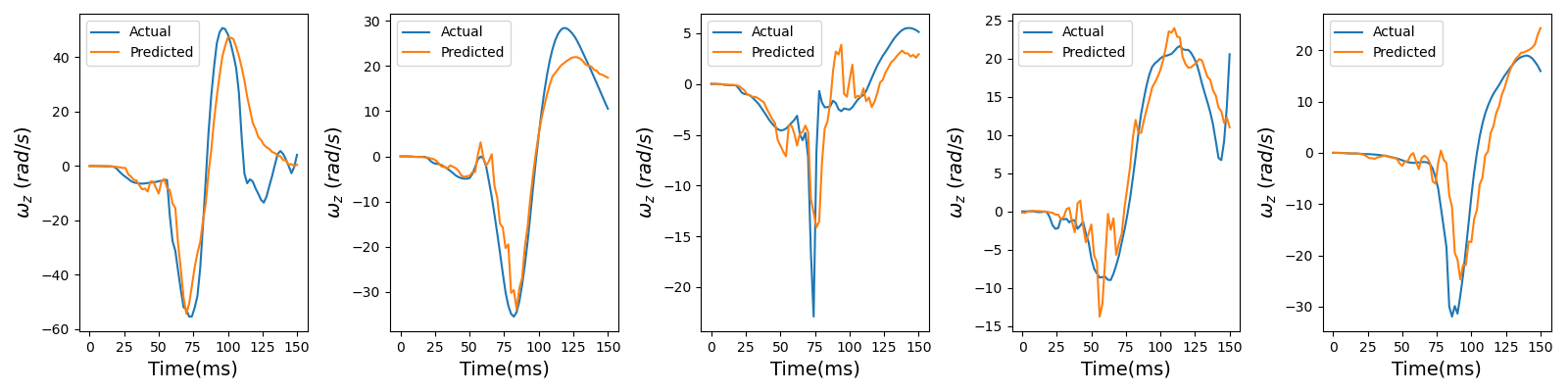  CORA=0.82  CORA=0.83  CORA=0.66  CORA=0.75  CORA=0.65 |
| **c)** |
| **Supplementary Figure S5.** Time history comparisons for a) ω_x_, b) ω_y_, and c) ω_z_ for 250 fps. |

| 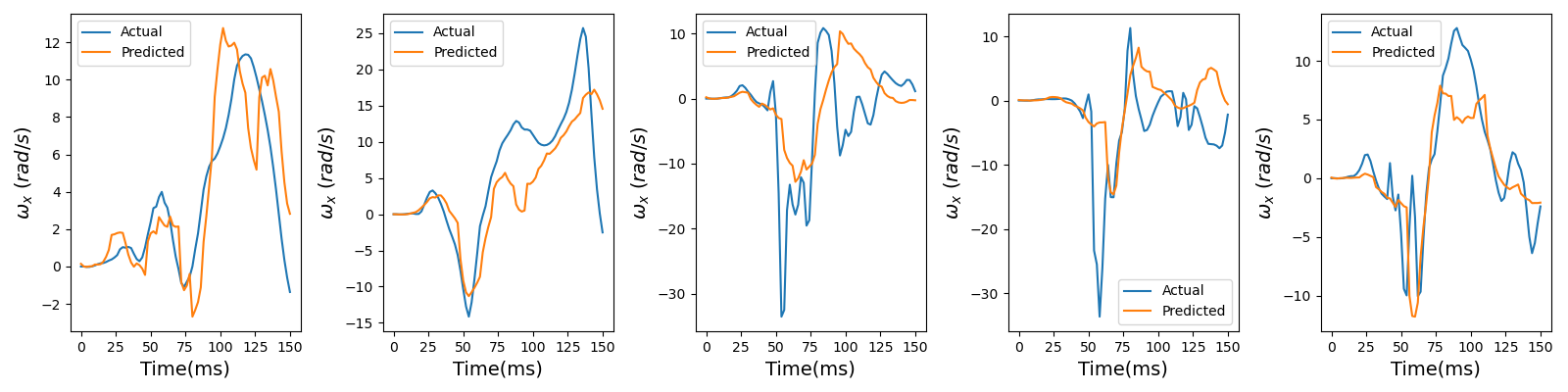  CORA=0.65  CORA=0.57  CORA=0.65  CORA=0.48  CORA=0.43 |
| --- |
| **a)** |
| 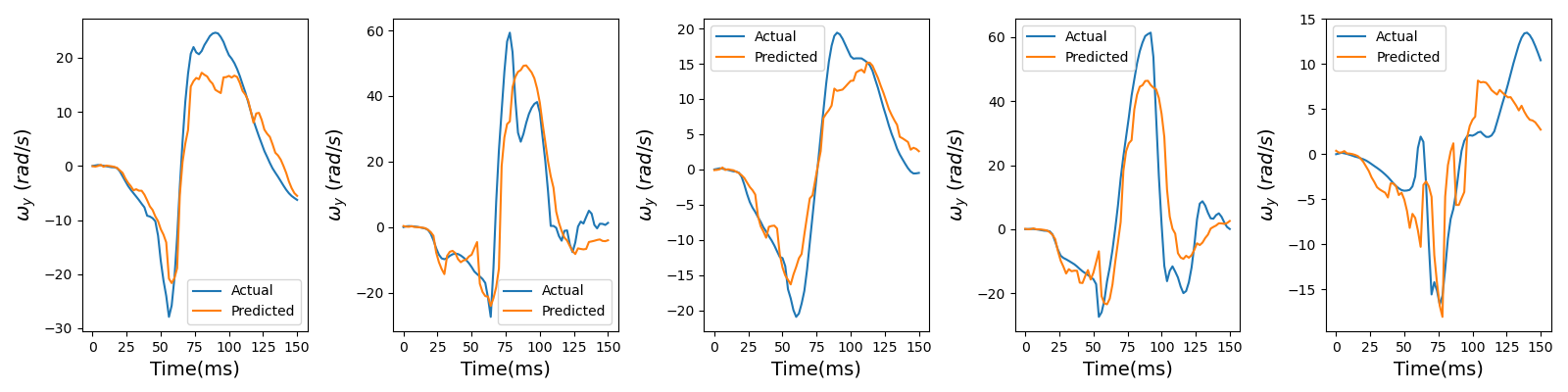  CORA=0.57  CORA=0.73  CORA=0.76  CORA=0.75  CORA=0.75 |
| **b)** |
| 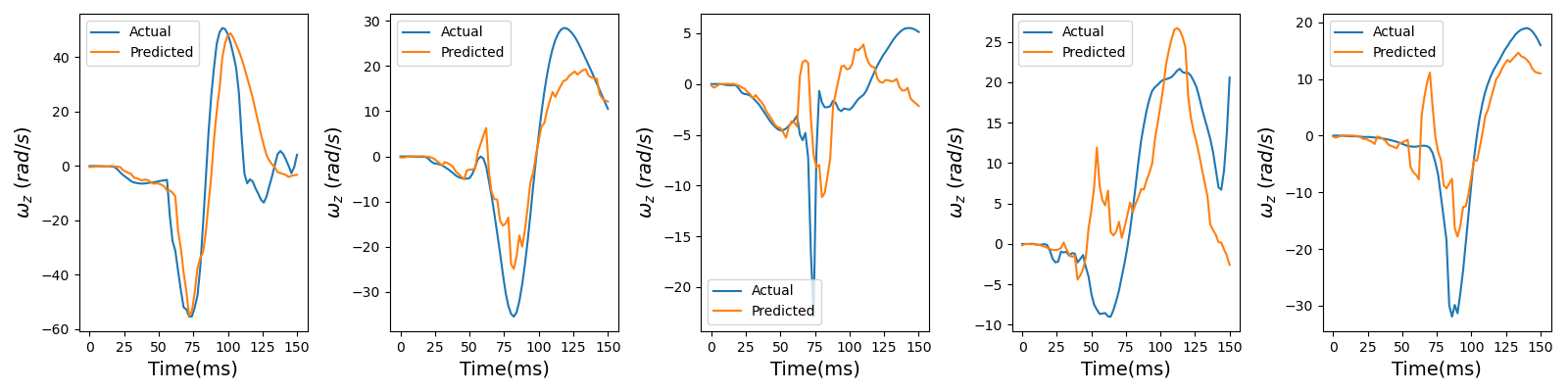  CORA=0.52  CORA=0.72  CORA=0.67  CORA=0.52  CORA=0.55 |
| **c)** |
| **Supplementary Figure S6.** Time history comparisons for a) ω_x_, b) ω_y_, and c) ω_z_ for 125 fps. |

| 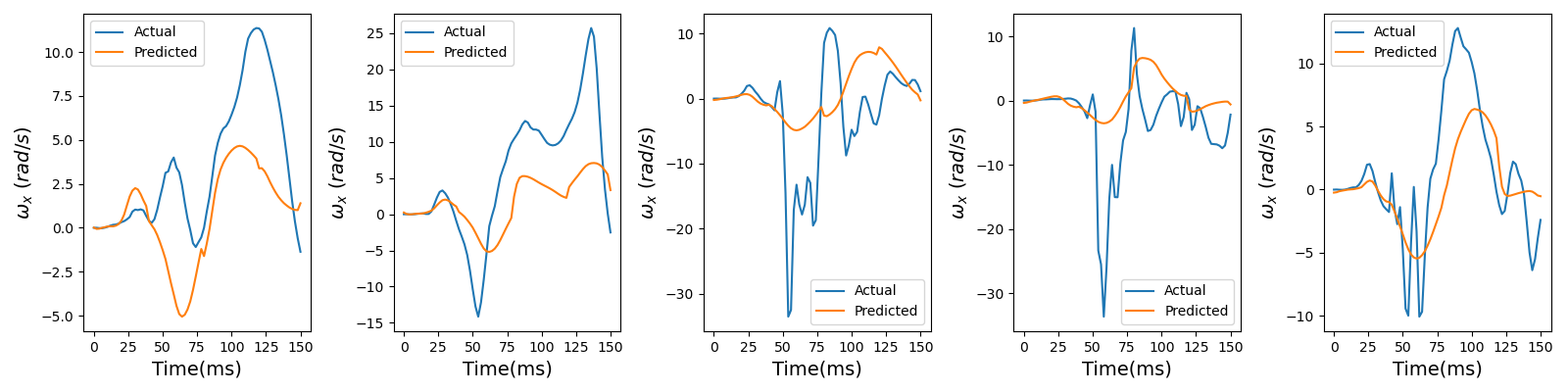  CORA=0.48  CORA=0.43  CORA=0.29  CORA=0.38  CORA=0.46 |
| --- |
| **a)** |
| 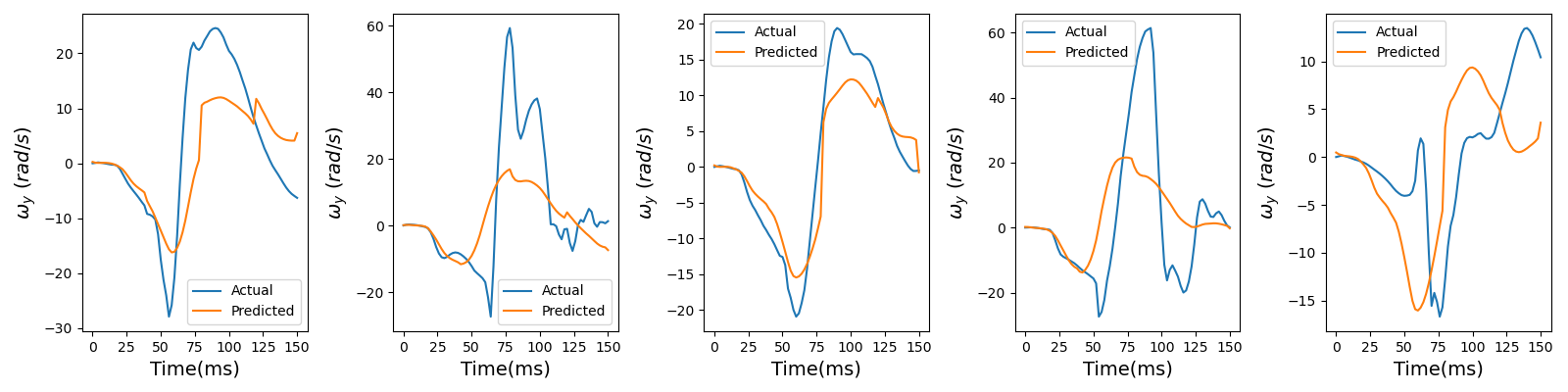  CORA=0.32  CORA=0.35  CORA=0.65  CORA=0.49  CORA=0.50 |
| **b)** |
| 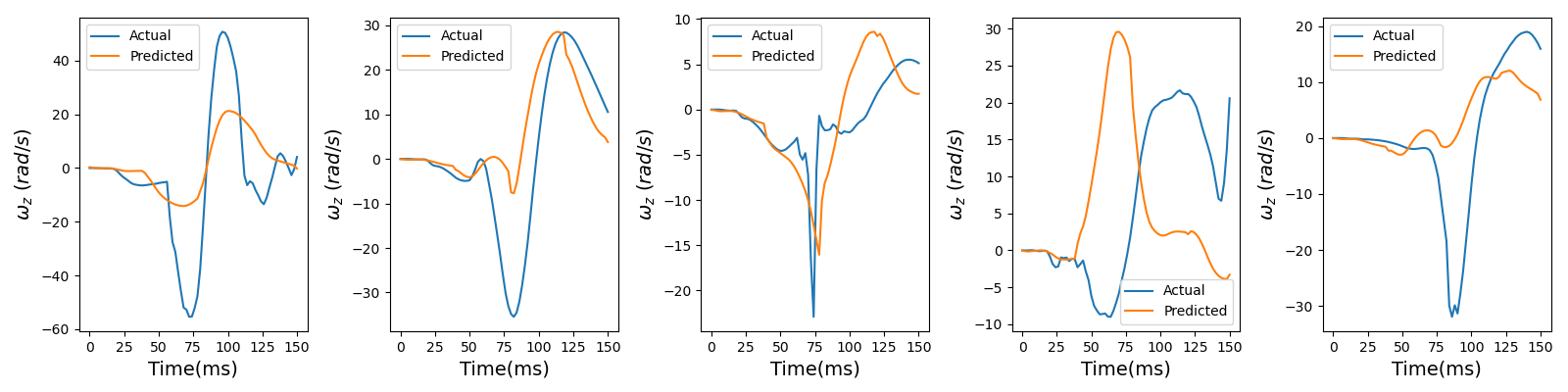  CORA=0.54  CORA=0.30  CORA=0.34  CORA=0.46  CORA=0.43 |
| **c)** |
| **Supplementary Figure S7.** Time history comparisons for a) ω_x_, b) ω_y_, and c) ω_z_ for 25 fps. |

**References**

1. Bradski, G. The OpenCV Library. *Dr Dobb’s Journal of Software Tools* (2000).
2. O'Malley, T., Bursztein, E., Long, J. & Chollet, F. Keras-Tuner (2019).
3. Snoek, J., Larochelle, H. & Adams, R.P. Practical Bayesian Optimization of Machine Learning Algorithms. *Advances in Neural Information Processing Systems* (2012).
